# Synthesis and Properties of Ba*M*TeS (M = Fe, Mn, Zn) and the Disordered Structural Analog BaGe_0.5_TeS

**DOI:** 10.1021/acs.inorgchem.4c00146

**Published:** 2024-06-06

**Authors:** Emil H. Frøen, Domenic Nowak, Peter Adler, Martin Valldor

**Affiliations:** † Centre for Materials Science and Nanotechnology (SMN), Department of Chemistry, University of Oslo, Sem Sælands vei 26, Oslo N-0371, Norway; ‡ Leibniz Institute for Solid State and Materials Research (IFW), Helmholtzstraße 20, Dresden 01069, Germany; § Max Planck Institute for Chemical Physics of Solids, Nöthnitzer Straße 40, Dresden 01187, Germany

## Abstract

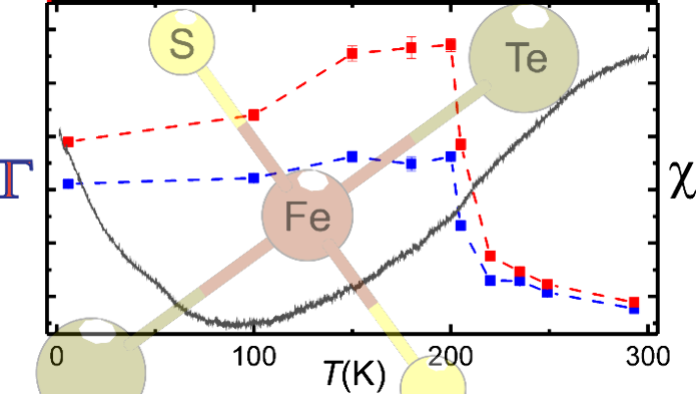

A series of tertiary sulfide-tellurides, Ba*M*_*x*_TeS (M = Fe, Mn, Zn, Ge), has been synthesized by solid-state synthesis. The compounds assume an orthorhombic crystal structure, described by the *Cmcm* (No. 63) space group, and are structural analogs of the Ba*M*SO (M = Co, Zn) phases. The properties of all four analogs are investigated by DFT analysis. As only the BaFeTeS analog was prepared as a relatively pure phase, this homologue was subject to further experimental investigations, including heat capacity, magnetometry, and Mössbauer spectroscopy. BaFeTeS exhibits no obvious phase transition between 2 and 300 K, has no paramagnetic behavior, and lacks long-range magnetic ordering. However, the Mössbauer spectra, as well as electrical resistance data, indicate a hidden transition near 200 K that is tentatively explained by a dynamic charge-density-wave mechanism, based on a resonating valence bond (RVB) model.

## Abstract 2

Four isostructural phases with general compositions Ba*M*TeS (M = Fe, Mn, Zn) and BaGe_0.5_TeS have been synthesized by simple solid-state synthesis. The compounds assume the crystal structure of Ba*M*SO (M = Co, Zn). The Fe analog was prepared as a sufficiently pure phase for further analysis and was found to exhibit an unexpected lack of magnetic ordering, as well as a Mössbauer spectroscopy anomaly at lower temperatures.

## Introduction

The ordering of anions in bichalcogenides,^1^ as part of the more general bianion systems,^2^ has proven a successful path toward novel chemistry and crystal structures. To avoid anion solid solutions, the ionic radii of the two anions need to differ by more than about 15%,^3^ leaving oxide-sulfides or sulfide-tellurides as probable systems to form anionic superstructures, as opposed to a solid solution. A direct consequence of an anionic superstructure is that the overall crystal structure acquires a larger periodicity with possibility for reduced dimensionality. Examples of this include 1D structures such as magnetic chains in La_4_MnS_6_O^4^ leaving oxide-sulfides or sulfide-tellurides as probable systems to form anionic superstructures, as opposed to and La_5_V_3_O_7_S_7_,^5^ spin-ladders in SrFe_2_*Ch*_2_O (*Ch* = S, Se),^6^ or 2D structures as in La_2_Fe_2_Ch_2_O_3_ (*Ch* = S, Se),^7,8^ and Sr_2_Fe_3_S_2_O_3_.^9^ One example of comparatively recent interest is the Ba*M*SO (M = Co, Zn) series.^10,11^ This structure type has been theoretically predicted to have potential for research on unconventional high-temperature superconductivity.^12^

Bichalcogenides, as a part of the more general field of multianions as a whole, have been rapidly gaining momentum in the scientific literature in recent years, continuously growing since the discovery of the high-temperature iron-based superconductors in 2008.^13^ Since then, multianions have shown potential in a range of fields, including catalysis and^14,15^ battery applications,^16^ and, perhaps most characteristically, tunable properties.^2^ Some compounds within the bichalcogenides specifically have gained attention for their potential in important practical applications. For instance, BiCuSeO has been identified as a promising candidate for thermoelectric materials.^17^ Other systems of interest include the many lanthanide oxysulfide phosphors,^18^ and numerous oxysulfides have been found to exhibit promising nonlinear optical properties.^19,20^ While there are a fair number of known oxysulfides, the number of known sulfide-tellurides is comparatively limited. As such, there is much to be discovered in investigating these phase diagrams. The chemistry of the relatively large telluride ion needs to be further explored.

Here, we present a new series of structural analogs to the Ba*M*SO compounds of the form Ba*M*TeS (M = Fe, Mn, Zn), as well as the disordered isostructural BaGe_0.5_TeS. We report on their synthesis, as well as the detailed properties of BaFeTeS.

## Experimental Section

### Sample Preparation

All handling of the samples was carried out inside an argon-filled glovebox (H_2_O <0.1 and O_2_ <1 ppm). For the pure Fe phase, a mixture totaling about 0.5 g with stoichiometric composition BaFeTeS was weighed out from BaS (Alfa Aesar, 99.7%), Fe powder (Alfa Aesar, 99%), and Te powder (Thermo Scientific, 99.99%). The components were mixed together into a homogeneous mass and pressed into pellets with a diameter of 13 mm with 35 kN of force. The resulting pellet was broken up into fragments, which were subsequently placed in corundum crucibles. The crucibles were inserted into silica ampules, which were evacuated and sealed with an oxygen–hydrogen torch after lowering the internal pressure of Ar to about 10^–2^ mbar.

The sample was heated at a rate of 5 K min^–1^ up to 873 K (the same rate was used for all heating cycles), where it was left to rest for 96 h, before being allowed to cool to room temperature at an ambient rate. During this initial reaction step, the elemental components of the precursors all reacted to form intermediate phases with significantly higher melting points. The subsequent heating periods at slightly higher temperatures were for the purpose of further reacting these intermediate phases to form the final product. The cooled sample was moved back into the glovebox, reground, pelletized, and sealed in an ampule, which was then heated to 923 K. The sample was left to rest at this temperature for 48 h, followed by another regrinding. Finally, the sample was heated to 933 K and left to rest at this temperature for 480 h, with four more regrindings to attain a pure phase. The final product was a highly crystalline, black phase; however, due to the low synthesis temperature, the pellet fragments exhibited poor sintering and a highly porous structure. The low synthesis temperature was necessitated by the fact that BaFeTeS constitutes the low-temperature phase of the Ba–Fe–Te–S phase system. Running the synthesis at a higher temperature risked irreversible formation of the intermediate temperature Ba_6_Fe_2_Te_3_S_7_^21^ phase or a yet unreported high-temperature phase in the same system.^22^

The synthesis of single crystals for the other three phases utilized the same BaS and Te sources. The transition metal element analogs used the same general procedure, with different metal elements and heating procedures. The metal elements used were Mn (Aldrich, ≥99.9%), Zn (≥99.9%), and Ge (Thermo Scientific, 5N). Additionally, the synthesis of the Ge analog single crystals utilized a prereacted mixture of 1:1 Ge:Te, prepared by heating under inert conditions at 1023 K for 48 h; however, the nominal stoichiometry was the same as for the other three phases (1:1:1:1). All syntheses used a heating rate of 5 K min^–1^. BaMnTeS crystals were formed by a single heating round at 1273 K for 48 h, but the obtained sample contains several secondary phases.

Crystal growth of BaZnTeS required heating to around 1273 K to obtain a congruent melt. The target phase formed upon cooling this melt down below the melting point (located in the 1173–1273 K range), forming a single large crystal lump with a vivid yellow color. Difficulties with the synthesis originated with the fact that zinc telluride would evaporate out of the melt, which resulted in nonstoichiometric compositions, as well as BaS contamination of the final product. Much of the BaS ends up covering the surface of the crystal lump, but some of this contaminant does remain within the bulk of the sample and cannot be easily removed. During the synthesis of BaGe_0.5_TeS, a similar problem as with the Zn variant occurred with the sublimation of GeTe. In this case, the loss of reactants resulted in the irreversible formation of Ba_3_GeTeS_4_.^23^ The single crystal was obtained with a single heating cycle at 973 K for 48 h.

No uncommon hazards are noted with the experimental work.

### X-Ray Structure Determination

Single crystal diffraction data (SC-XRD) were obtained using the Bruker D8 Venture single crystal diffractometer, with a Mo*K*α InCoatec microfocus X-ray source and Photon 100 detector. Room-temperature structural determinations were carried out on all phases, while BaFeTeS was additionally measured at 100 K. For the measurement of the powder X-ray diffractograms (PXRD), a Bruker D8 Discover diffractometer in Bragg–Brentano geometry was used. The instrument used Cu*K*α_1_ X-rays with a Ge(111) Johanssen monochromator and a Lynxeye detector. The sample was mounted upon a zero-background-oriented silicon plate, with a small quantity of silicon grease to fix the powder in place. Structure solution and refinement were carried out with the JANA2020 software.^24^

### Physical Property Measurement System (PPMS)

The determination of the physical properties of BaFeTeS was done using a Quantum Design PPMS. DC magnetic susceptibility measurements were carried out on a sample taken from a powdered pellet, contained within a polypropylene sample holder. Field-Cooled (FC) and Zero-Field-Cooled (ZFC) measurements were performed over the 2–300 K range, with applied fields of 100 mT and 1 T. Heat capacity measurements were carried out over the same temperature range, utilizing the nonadiabatic thermal relaxation method. The sample was allowed to rest for 5 min at each temperature to equilibrate prior to each measurement. A larger quantity of coupling grease was used for the measurements, in which small sample pieces were immersed, as the porosity of the pellets caused difficulties. As a result, the sample coupling was not ideal.

The electrical resistance measurements were carried out using the same PPMS system to control the temperature. To measure the electrical resistance, a MASTECH MAS830L multimeter with two contacts was used. The contacts were connected to a sintered pellet with the aid of silver paint. The resistance was measured during constant ramping of the temperature by 5 K min^–1^ between 10 and 300 K and 1 K min^–1^ between 2 and 10 K. For analysis, the average value between heating and cooling ramps was used. The resistance at 2–3 K was beyond measurement capabilities (>2 MΩ) and is thus not included in the data.

### Scanning Electron Microscopy (SEM) and Energy-Dispersive X-Ray (EDX) Analysis

The SEM imaging utilized a Hitachi SU8230 field emission scanning electron microscope, with an XFlash 6|10 EDX detector for elemental analysis. An acceleration voltage of 15 keV was set for both imaging and elemental analysis. To account for the limited accuracy of EDX for quantitative determination of chemical compositions, averaged values from elemental analysis of 20 separate crystallites for each species were used to determine the final EDX composition. The barium content was used as the reference point for the rest of the composition, with other elements normalized accordingly.

### Mössbauer Spectroscopy

^57^Fe Mössbauer spectra were collected between 293 and 6 K employing a standard WissEl spectrometer operated in constant acceleration mode with a ^57^Co/Rh source. The temperature control used a Janis SHI 850-5 closed cycle refrigerator. About 33 mg of powdered BaFeTeS was dispersed in BN and distributed in an acrylic glass sample container with a 13 mm inner diameter. Isomer shifts are reported relative to α-iron. The data evaluation utilized the MossWinn program,^25^ assuming the thin absorber approximation.

### Density Functional Theory (DFT)

DFT was used to investigate the electronic properties and, where applicable, the magnetic configurations of the four analogs. The calculations utilized the Vienna *ab initio* simulation package (VASP),^26,27^ with the generalized gradient approximation (GGA) Perdew–Burk–Ernzerhof (PBE)^28^ functional for the exchange-correlation energy. Projected augmented-wave (PAW)^29^ pseudopotentials were used, with a plane-wave cutoff energy of 600 eV. The convergence criteria for the self-consistent-field energy and ionic relaxation were 10^–6^ eV and 0.01 eV Å^–1^, respectively.

To account for the strong correlation of the *d*-orbital electrons, the Hubbard +U approach was employed under the rotationally invariant Dudarev approach.^30^ A full range of calculations with varying *U*_eff_ values were carried out for the *d*-elements, with *U*_eff_ = 0–6 eV, to observe how the systems behave under varying parameters. The calculations for the magnetic analogs with Fe and Mn were carried out using a 2 × 1 × 1 supercell, as this was necessary to represent the magnetic structure. For structural relaxation calculations, the sampling of the Brillouin zone utilized a gamma centered, 3 × 2 × 4 grid. The BaZnTeS analog utilized a minimal representation calculation cell, with a 6 × 6 × 6 sampling grid. Finally, the partially occupied Ge analog utilized a minimal representation calculation cell corresponding to an ordered 0.5 occupancy within a 2 × 2 × 2 supercell of the basic unit cell, utilizing a 6 × 6 × 5 sampling grid. Calculations of the density of states (DOS) utilized a doubled grid for sampling the Brillouin zone, compared with the structural relaxation. Determination of the symmetry path for the band structure was done with the SeeK-path tool.^31,32^ Integration over the Brillouin zone for all calculations utilized the tetrahedron method with Blöchl corrections and a smearing width of 0.02 eV. Larger supercells of the Fe analog as well as noncollinear magnetic calculations with spin–orbit coupling were attempted while searching for indications of Peierls distortion or similar effects in the Fe analog. Finally, it should be noted that, while the Mn analog appears to be nonstoichiometric, the calculations utilize fully occupied, ordered cells.

## Results

### Crystal Structure

**Figure 1:**
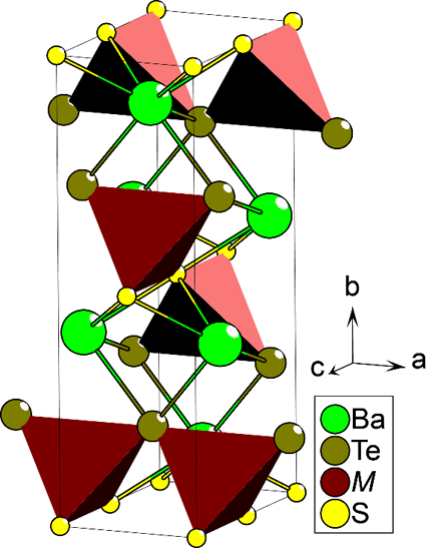
Crystal structure shared by the Ba*M*TeS phases (M = Fe, Mn, Zn, Ge).

The compounds all crystallize in an orthorhombic crystal structure described by the *Cmcm* (No. 63) space group. Figure 1 shows the full crystal structure, which is isostructural with the previously known oxysulfide analogs Ba*M*SO (M = Co and Zn) phases.^10,11^ The refinement data and lattice parameters of the Ba*M*TeS crystal structures, as determined by SC-XRD, are given in Table 1. For detailed information on atomic positions and related thermal parameters, please refer to the Supporting Information.

**Table 1:** Refinement Details of the Ba*M*TeS Phases

formula	BaFeTeS		BaMnTe_0.86_S_1.14_	BaZnTeS	BaGe_0.5_TeS
radiation	Mo *K*α (λ = 0.71073 Å)				
instrument	Bruker D8 Venture				
crystal system	orthorhombic				
space group	*Cmcm* (No. 63)				
physical appearance	black		red-orange	yellow	dark red
temperature/K	100	293	293		
formula weight/g mol^–1^	352.85		338.56	362.38	333.29
*a*/Å	4.4463(3)	4.4275(7)	4.4947(3)	4.378(1)	4.5496(5)
*b*/Å	14.194(1)	14.196(2)	14.537(1)	14.528(3)	13.895(2)
*c*/Å	7.0780(5)	7.075(2)	7.1413(5)	7.092(2)	7.0371(9)
*V*/Å^3^	446.69(5)	444.7(2)	466.60(6)	451.1(2)	444.87(9)
*Z*	4				
ρ_calc_/g cm^–3^	5.2467	5.2704	4.8196	5.3363	4.9763
independent reflections	936	282	679	546	664
no. of variables	17	17	17	17	17
GOF (obs)	1.83	1.84	1.90	1.70	1.27
GOF (all)	1.71	1.69	1.83	1.48	1.19
R1 (obs)/%	2.62	3.01	3.14	4.33	2.95
R1 (all)/%	4.02	6.26	3.91	9.92	5.62
wR2 (obs)/%	6.40	7.03	7.62	7.40	6.17
wR2 (all)/%	6.65	7.63	7.77	8.36	6.91
CCDC ID	2309979	2309978	2309980	2309982	2309981

From simple visual inspection, the colors of the Ba*M*TeS analogs suggest that the phases exhibit increasing band gaps in the order Fe, Ge, Mn, and Zn. For the transition metal elements, this corresponds with the common trend observed for compounds of these elements. Visual indications of nonlinear optics were observed in impure samples of the BaZnTeS phase, but this has not been confirmed by optical measurements and remains a tentative possibility.

While the compounds assume a known crystal structure, we will emphasize the structural environment of the metal cations for further discussion later. The *M* positions are situated in a 2 + 2 heteroleptic, tetrahedral coordination. The coordination is anisotropic, with the *M*–Te–*M* and *M*–S–*M* coordinations being arranged along the *a*- and *c*-axes, respectively, forming continuous chains with this single bridging anionic species. Parallel with the *a*-axis, adjacent *M*-tetrahedra are connected exclusively through vertex-sharing telluride positions, featuring an *M*–Te–*M* angle of about 115°—although there is some variation between analogs. Along the *c*-axis, the *M*-tetrahedra are connected only via vertex-sharing of the sulfide positions, with a *M*–S–*M* angle of 180°. Each *M*-tetrahedron is thus coupled with four adjacent *M*-positions, resulting in an interconnected 2D network, which extends along the *ac*-plane. Adjacent *M*-layers along the *b*-axis are arranged with relative offsets of 0.5 along the *a*-axis. Due to this relative positioning, each *M*-position is situated equidistantly from its four closest neighbors in the adjacent *M*-layer.

The Mn variant appears to exhibit significant nonstoichiometry at the telluride positions. The partial substitution values of Te with S in the BaMnTeS crystal structure are based on refining the Te occupancies, as a full occupancy was found to result in an inadequate fit. The choice to substitute the missing Te with S was made based on the EDX data—explained in a later section—and on the basis that Mn is unlikely to assume a different oxidation state than Mn^2+^. The given composition of Te and S, BaMnTe_0.86_S_1.14_, was obtained by rounding the refined occupancies to two decimal places and locking the occupancy to these values. Uniquely among the four, the germanium analog appears to exhibit a disordered half occupancy, which likely relates to the relative stability of the 4+ state, relative to that of the 2+. We did not find any evidence to indicate that the occupancy of the Ge-sites is arranged with any form of long-range order.

Low-temperature measurement of the BaFeTeS crystal structure exhibits, within the precision of SC-XRD, the same space group, unit cell, and atomic positions as the room-temperature measurement. For the sake of later discussion, we will mention here that the thermal parameters of the Fe positions are the most anisotropic within the crystal structure, showing a distinct elongation along the *a*-axis. This same trait occurs in both the 293 and 100 K measurements, although the latter has a significantly smaller magnitude of the thermal parameters, as would be expected. In the case of the Mn analog, this elongation is even more pronounced, whereas for the Zn and Ge analogs, it is much less pronounced and effectively nonexistent, respectively.

### Powder XRD

**Figure 2:**
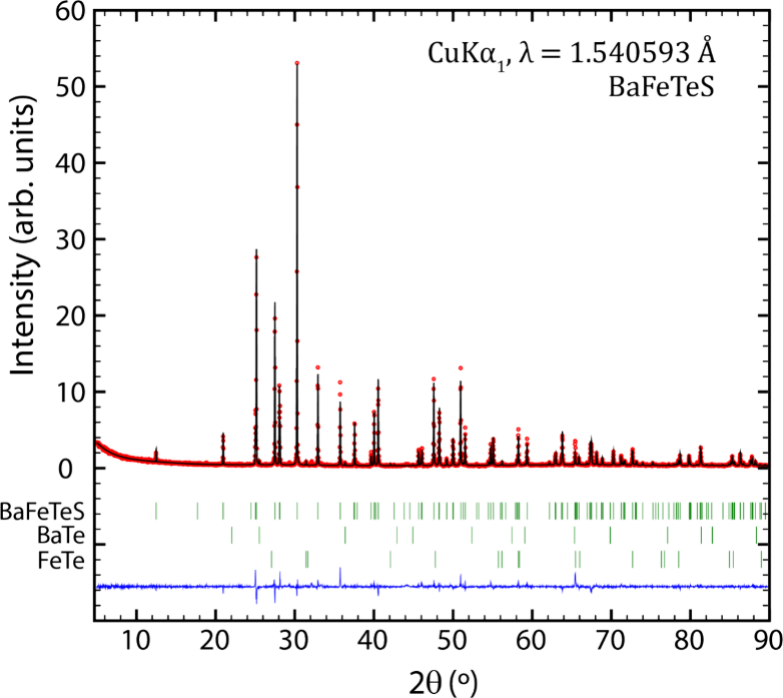
Rietveld refinement of the PXRD of the BaFeTeS sample. The red circles are observations, the full black line is the Rietveld refinement, the vertical lines are Bragg positions of the phases indicated on the left, and the blue line is the difference between the observed and calculated intensity values.

To obtain a more accurate value for the BaFeTeS lattice parameters, PXRD was utilized. Refining the lattice parameters by Rietveld, the values obtained are *a* = 4.45517(3) Å, *b* = 14.26727(9) Å, and *c* = 7.09845(4) Å. The PXRD data, shown in Figure 2, show secondary phases present, including BaTe and FeTe,^33,34^ but these constitute a minor proportion; the two identified secondary phases together constitute less than 1 vol % of the product. There is at least one unidentified secondary phase, with a PXRD signal of comparable magnitude to BaTe and FeTe.

### SEM and EDX Analysis

**Figure 3:**
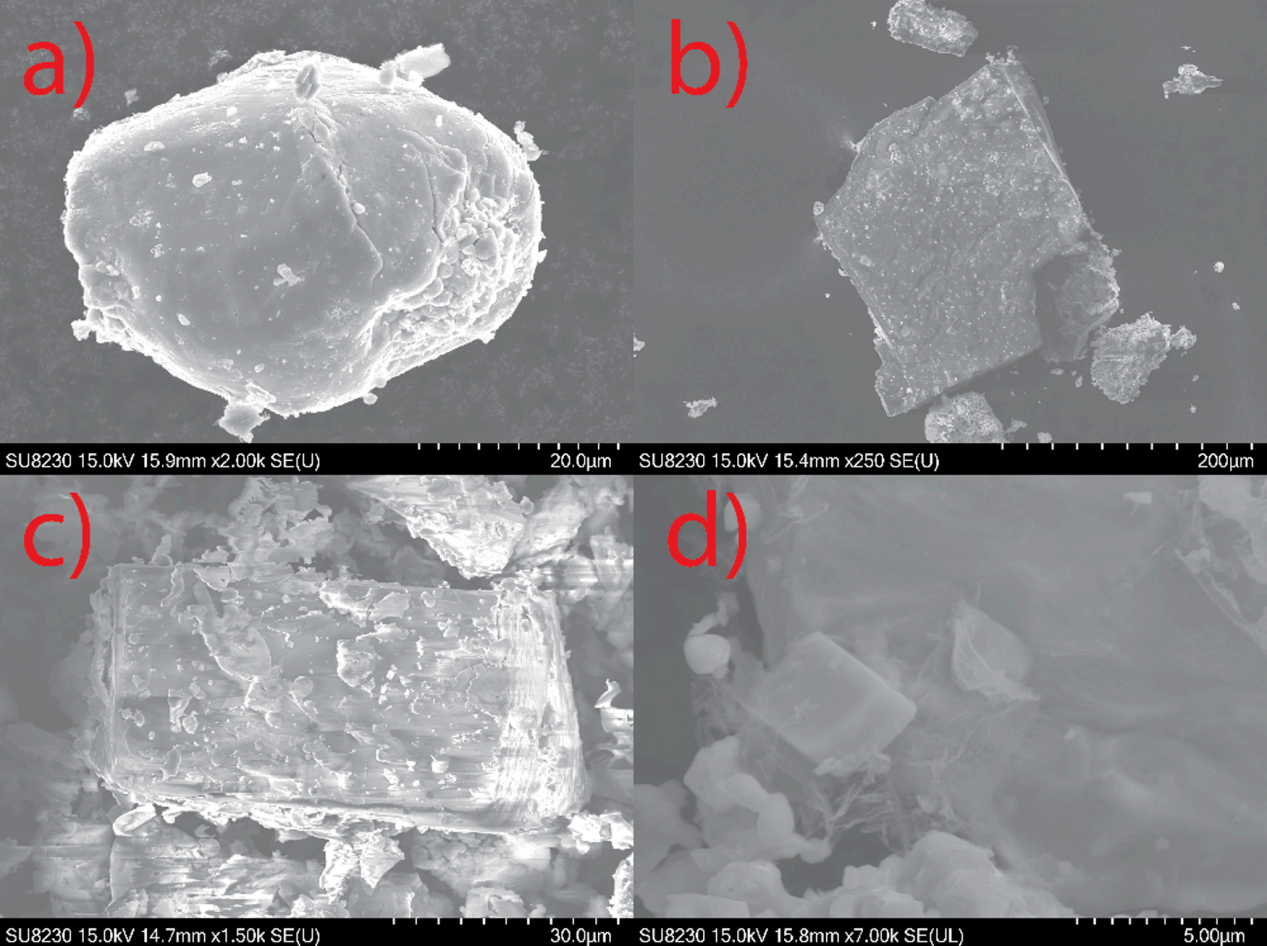
SEM image of (a) BaFeTeS, illustrating the amorphous-looking structure. (b) BaMnTeS. (c) BaZnTeS, illustrating the rectangular prismatic crystals and layered structure, the latter observed on the right face. (d) BaGe_0.5_TeS, illustrating the filament-like structures associated with the crystals.

SEM images of the four phases are shown in Figure 3. While three of the phases exhibit distinct crystals, the Fe analog (Figure 3a) forms amorphous-looking structures, with rounded appearances. The Mn (Figure 3b) and Zn (Figure 3c) analogs both exhibit standard, rectangular prismatic crystals. The Ge (Figure 3d) analog exhibited an unusual feature: the crystals were always accompanied by structures resembling thin, cotton-like structures on and around the bulk crystals. The nature and composition of these cotton-like structures are unknown. The elemental composition of the four analogs, determined by EDX analysis, is shown in Table 2.

**Table 2:** Elemental Composition of Ba*M*TeS, as Determined by EDX Analysis^a^

compound/element	Ba	*M*	Te	S
BaFeTeS	1.00(5)	1.12(5)	0.97(3)	1.07(4)
BaMnTeS	1.00(1)	1.08(3)	0.90(5)	1.11(5)
BaZnTeS	1.00(2)	1.10(3)	1.00(3)	1.01(3)
BaGe_0.5_TeS	1.00(2)	0.49(5)	1.02(3)	0.97(3)

a Normalized according to the Ba composition.

The averaged values of the Ba*M*TeS analogs show an excess of the metal elements Fe, Mn, and Zn, relative to the nominal stoichiometry. The Mn analog exhibits a significantly substoichiometric content of Te, counterbalanced by a superstoichiometric proportion of S. The EDX results thus indicate that the Te-positions in BaMnTeS are partially occupied by sulfur. The germanium analog showed the elemental composition expected from the SC-XRD measurement, corresponding with a half occupancy of Ge in the structure. During EDX measurements of the Ge analog, an unusual observation was made, which is elaborated upon in the Supporting Information.

### Heat Capacity

**Figure 4:**
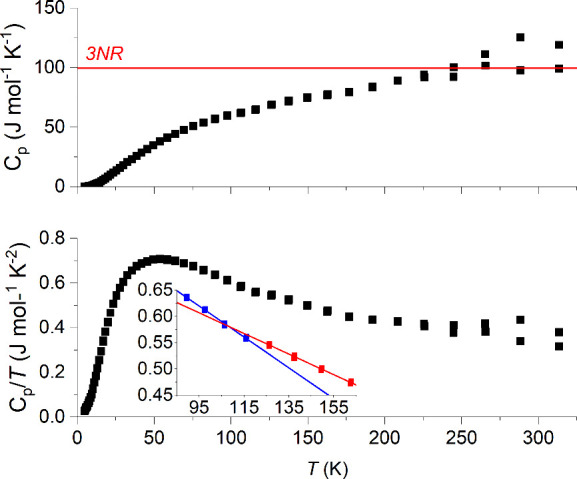
Heat capacity of BaFeTeS with temperature. At each temperature, two measurements were done, and both are shown for the sake of reproducibility. The lower inset emphasizes the irregularity in the curve: each line is fitted to the data points of the same color.

The heat capacity measurement of BaFeTeS is shown in Figure 4. Due to the low quality of the sample for purposes of heat capacity measurement, the results are not ideal, especially at high temperatures. In addition to the noise at higher temperatures, the heat capacity exceeds the Dulong–Petit limit. There is an irregularity in the heat capacity around the 105–135 K range, but whether this is an intrinsic effect or not is unknown; it is possibly an artifact of the larger quantity of grease used to connect the sample with the holder. While the data quality is insufficient to determine the presence or absence of higher-order transitions with certainty, there does not appear to be a first-order transition over the measured temperature range.

### Mössbauer Spectroscopy

**Figure 5:**
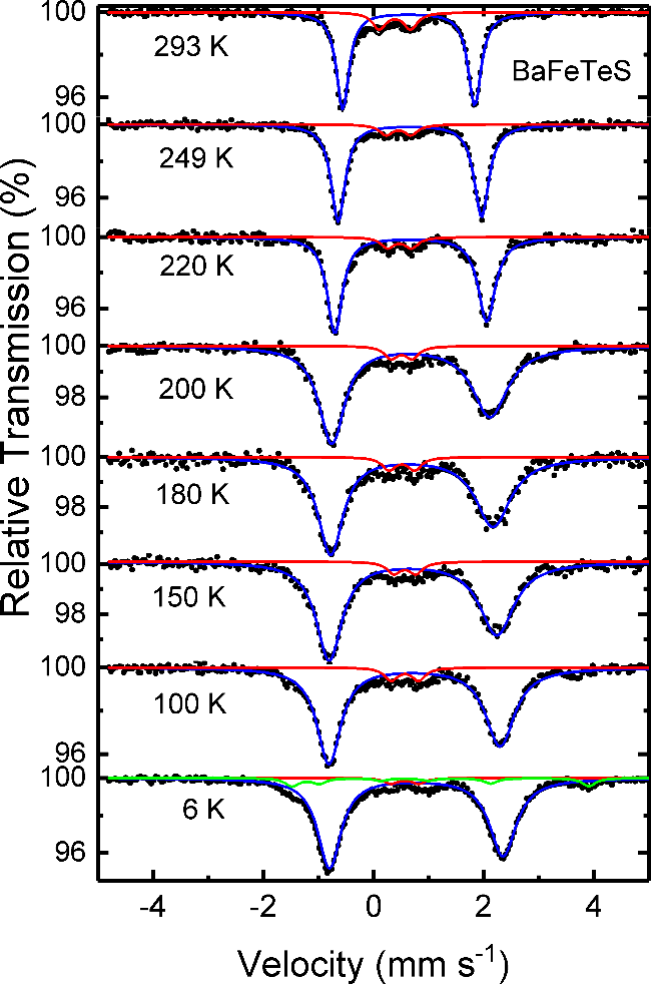
Temperature dependence of the Mössbauer spectra. Dots, black lines, and colored lines correspond to the experimental data, the calculated spectra, and the component spectra, respectively. The small signals are attributed to impurities.

The temperature dependence of the Mössbauer spectra of BaFeTeS is shown in Figure 5. While the spectra feature a simple quadrupole doublet above ∼220 K, they become strongly broadened and exhibit an asymmetry of line-widths near 200 K. To describe the shape of the spectra, the fitting included a single doublet with uncorrelated line-widths over the whole temperature range. Note, in addition to this main component, additional weak signals are present at all temperatures. At low temperatures, a minority magnetically ordered phase is apparent, but, owing to the limited statistics of the spectra, it cannot be elucidated at which temperature this starts to appear. The minority signals are attributed to impurities. The doublet feature of the main component persists down to 6 K, verifying that BaFeTeS does not assume long-range magnetic ordering within the temperature range of this study.

**Figure 6:**
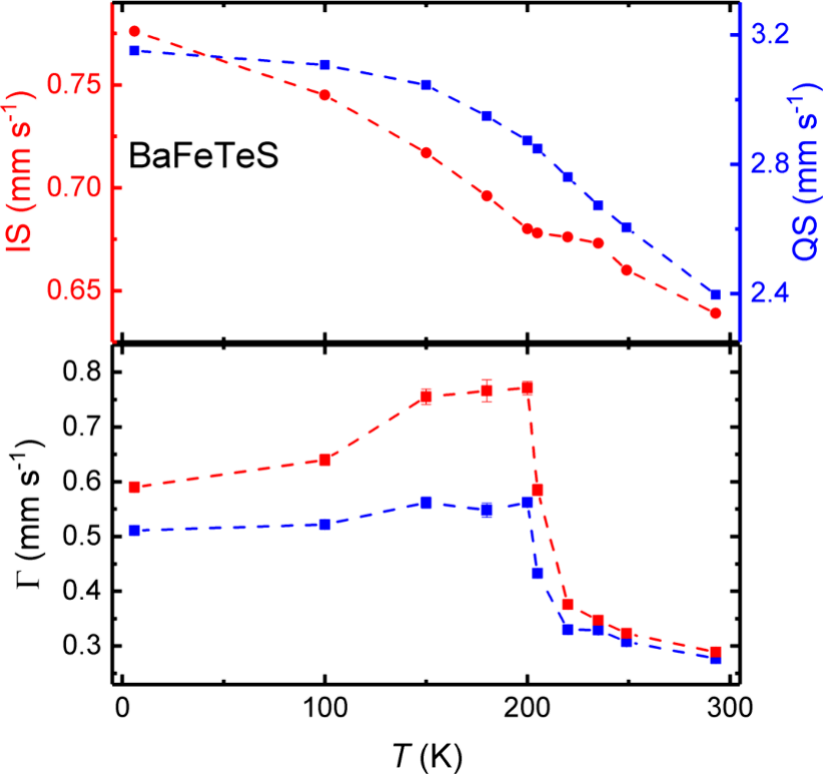
Results obtained from fitting the main component in the Mössbauer spectra by a doublet with independent line-widths. Top: temperature dependence of the isomer shift (red) and quadrupole splitting (blue). Bottom: temperature dependence of the line-width of the low-velocity (blue) and high-velocity (red) components of the doublet, respectively.

In Figure 6, the temperature dependence of the isomer shift (IS) and quadrupole splitting (QS) as well as of the line-widths (Γ) is depicted. The large IS values are consistent with Fe^2+^ in an approximately tetrahedral environment.^35^ This is in agreement with the pronounced temperature dependence of QS reflecting the *T* dependent population of the d orbitals of the Fe^2+^ ions that are split by the lower symmetric ligand field. The *T* dependence of IS mainly reflects the second-order Doppler shift; however, the data indicate an anomaly between 200 and 220 K, which is just the *T*-range where the spectra start to become asymmetric. For *T* ≤220 K, broadening of lines sets in, and an asymmetry of line-widths emerges, which is largest near 200 K and is somewhat reduced at lower temperatures. The low-temperature spectra, down to 6 K, remain much broader as compared to the high-temperature spectra.

In the following, possible reasons for the broadening and asymmetry of the spectra below 220 K are considered. Asymmetric doublets can arise from an anisotropy of the Lamb Mössbauer factor (Goldanskii-Karyagin effect, GKE)^36,37^ that is due to the anisotropy of the vibrational properties and leads to unequal line intensities. However, the most prominent feature in the present spectra is an asymmetric line broadening, which is not typical for the GKE.^38^ Furthermore, a GKE would not explain why the spectra become more symmetric in the high-*T* region. The asymmetric peak broadening could either have a static or dynamic origin. The former case implies a locally varying electronic and/or structural environment. In fact, the main features of the spectra could be reproduced by fitting them assuming a correlated distribution in IS and QS (Figure S1). Considering the low-dimensional structural features, a variation in the local environment could be caused by a charge-density-wave (CDW) and/or a Peierls distortion, but no evidence of such was observed in the low-temperature SC-XRD measurement.

BaFeTeS does not develop long-range magnetic ordering, but low-dimensional magnetic coupling, suggested by the magnetic susceptibility measurements (see Magnetism), could result in spin fluctuation processes and asymmetric line-widths (Blume-type asymmetry).^39,40^ However, in that case, it is expected that the spin dynamics slows down with decreasing temperature, and that the spectra evolve toward a static hyperfine pattern, which is clearly not observed. Another possibility would be a fluctuation between two electronically and/or structurally different configurations characterized by different IS and QS values, which also can be the origin for asymmetric doublets.^41^ Tentative simulations confirm that the shape of the present spectra can be generated by such a fluctuation process but this would imply configurations with a considerably different local environment.

The asymmetric line broadening evolves in a quite narrow *T*-range near 200 K, and the doublet feature persists down to low temperatures. It is not easy to understand these observations within a dynamic scenario, and thus a static explanation, suggesting a hidden electronic/structural transition near 200 K, appears more reliable. While the origin of the change in the spectra cannot be resolved unambiguously, it is remarkable that it occurs in the same *T*-range where also an anomaly in the electronic transport behavior is observed (see Electrical Resistance).

### Magnetism

To preface, the magnitude of the susceptibility of BaFeTeS is very low across the measured temperature range. Diamagnetic contributions from atomic sources and errors associated with the PPMS holder are significant at this scale. The qualitative trend is correct, but the absolute magnitude of the susceptibility is higher than the data suggest.

**Figure 7:**
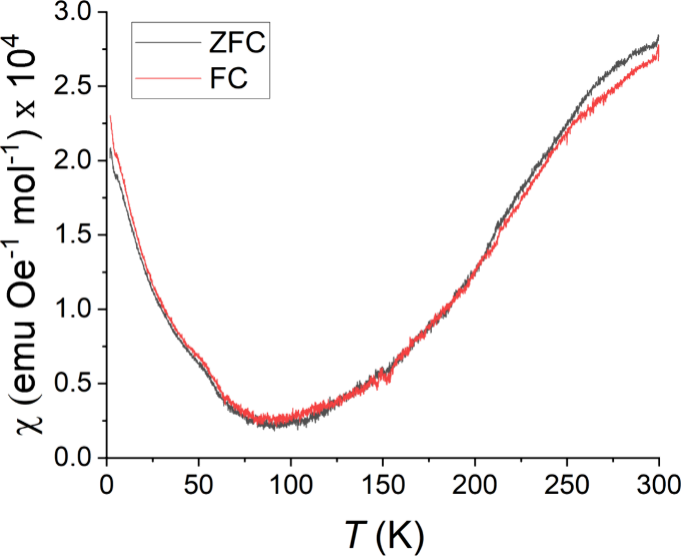
FC-ZFC DC magnetic susceptibility of BaFeTeS, from 2 to 300 K, with a 1 T external applied magnetic field.

BaFeTeS exhibits an unusual DC susceptibility trend in the 2–300 K range, with only slight differences between FC and ZFC (Figure 7). The measurements at 100 mT and 1 T exhibit the same qualitative features; however, only the data at 1 T are presented because they exhibit less noise. The susceptibility clearly does not exhibit regular Curie paramagnetism, but rather a trend of increasing susceptibility with temperature. With the results of the Mössbauer spectroscopy ruling out the possibility of a magnetic state with long-range order, it is notable that the observed trend is markedly similar to that of BaCoSO.^11^ The lack of both paramagnetic and ordered behavior in BaFeTeS suggests that a thermal activation mechanism of electron states could be the cause of the observed susceptibility behavior. An example of such behavior occurs, for instance, in the related Ba_6_Fe_2_Te_3_S_7_ phase,^21^ although the Fe structure of Ba_6_Fe_2_Te_3_S_7_ is dimeric, rather than planar as in BaFeTeS.

If one assumes that the Fe-layers are internally coupled in an AFM arrangement, a possible explanation for the observed behavior is that magnetic coupling between the Fe–S–Te layers may be geometrically frustrated, which could prevent long-range magnetic ordering from evolving. This possibility arises as a consequence of the four closest Fe positions between two different Fe-layers being equidistant. If each layer assumes an internal AFM arrangement, there is no spin configuration, which allows for interplanar coupling, without both parallel and antiparallel spin alignments.^42,43^ However, the structural analog BaCoSO exhibits magnetic ordering at a high temperature of 222 K.^11^ As such, it should be possible for BaFeTeS to assume an ordered state, yet it fails to do so down into the single-digit kelvin range. This could potentially be attributed to the Fe^2+^ ions assuming a more isotropic, Heisenberg-like spin-state, possibly due to the lesser difference in electronegativity between S and Te, relative to the difference between O and S.

The DC susceptibility shows an increase at the lowest temperatures. This is commonly observed in iron-based syntheses and is typically assigned to paramagnetic impurities. Paramagnetic impurity effects are likely present and constitute part of the susceptibility at the lowest temperatures. It is also possible that magnetic fluctuations within the material diminish with the lowering temperature, and the spins are slightly aligned with the external field.

### Electrical Resistance

**Figure 8:**
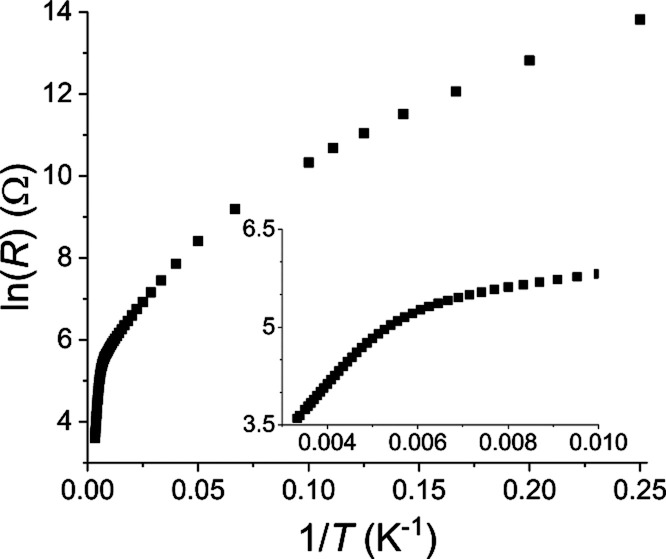
Arrhenius plot of the electrical resistance with temperature. The inset shows the high temperature, as well as the broad transition regions.

The electrical resistance of BaFeTeS, arranged as an Arrhenius plot, is shown in Figure 8. This plot shows that the electrical resistance of BaFeTeS may be divided into two distinct regions, with a broad intermediary temperature range, a high-temperature region, which exhibits a linear ln(*R*)–*T*^–1^ relation between 300 and ∼220 K, a broad transitional region between ∼220 and ∼130 K where the increase in d*R*/d*T* with lowering temperature slows and briefly plateaus (190–130 K), and a low-temperature region below 130 K. The final low-temperature region exhibits a sublinear ln(*R*)–*T*^–1^ relation. The linear relation in the high-temperature region indicates that BaFeTeS acts as a regular semiconducting material, with thermally activated charge carriers, in this temperature range. Deriving the band gap of BaFeTeS from a linear fit of the high-temperature region, the result is a narrow band gap of 136(1) meV. Below the transition temperature, the nonlinear relation precludes direct comparison, but the decreased gradient of the curve signifies that the effective activation energy for conduction of charge carriers has decreased, with the introduction of a presently unknown mechanism. Notably, the temperature regions of the electrical resistance closely correlate with the observed behavior from the temperature-dependent Mössbauer spectroscopy. The onset of the transitional region in the electrical resistance correlates with the emergence of the asymmetry and broad peaks in the Mössbauer data, while the transition to the low-temperature state occurs close to the temperature where the asymmetry starts to significantly diminish.

### Density Functional Theory (DFT)

In general, the calculated lattice parameters of the four analogs match well with the experimental lattices for all values of + *U*_eff_, with errors less than 4%, including those for Ge. A full occupation structure of the Ge analog, however, resulted in a 20% lattice error. All analogs are predicted to exhibit band gaps, albeit of differing character. As previously mentioned, between the four analogs available, their experimental color suggests the band gaps should lie, in increasing order, Fe, Ge, Mn, Zn. Comparing the GGA results without an applied Hubbard’s potential, this qualitative trend is indeed reproduced. Further details on the properties of the Mn, Zn and Ge analogs, as determined by DFT, are given in the Supporting Information. Only the Fe analog will be considered in depth here.

**Figure 9:**
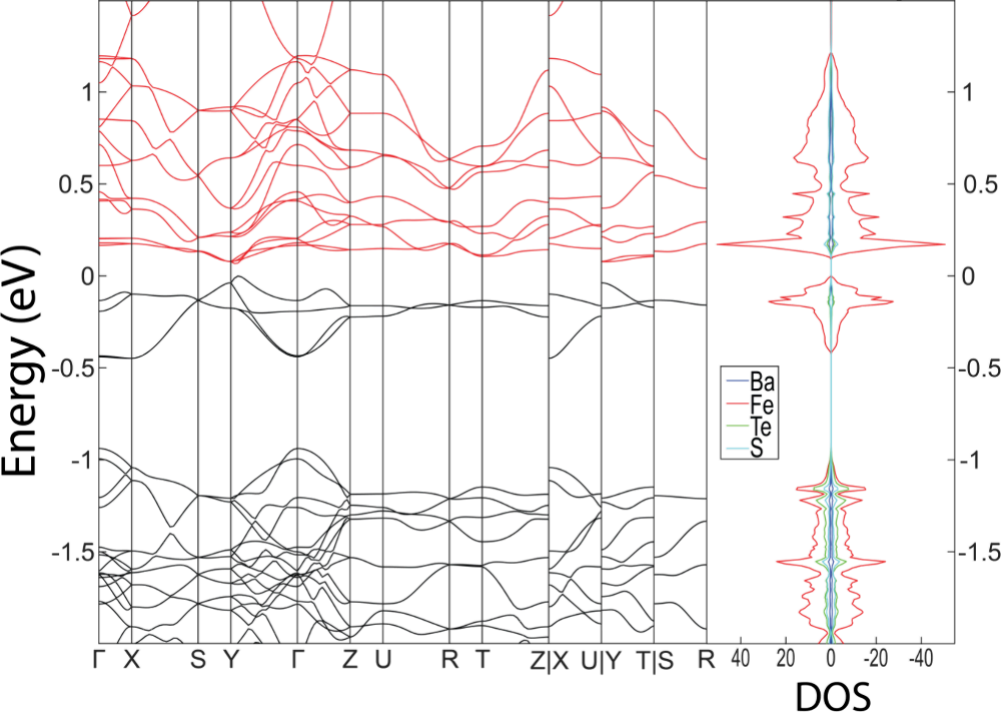
Band structure and DOS of BaFeTeS with *U*_eff_ = 0 eV. The black lines in the band structure correspond to valence bands, while the red lines correspond to conduction bands.

The calculations showed that the most favorable magnetic arrangement of BaFeTeS (and BaMnTeS), within a collinear framework, was an antiferromagnetic arrangement identical to the previously reported most favorable arrangement of BaCoSO.^10^ However, as BaFeTeS experimentally does not order, there is evidently some critical difference from the BaCoSO phase. The Fe analog is predicted to be a semiconductor for all values of *U*_eff_; however, at lower values, the band gap is very small. With *U*_eff_ = 0 eV, BaFeTeS is predicted to exhibit an indirect band gap of 69 meV, which is remarkably close to the high-temperature experimental value (136(1) meV). The band gap transition is found to occur at an off-symmetry position, with the full band structure shown in Figure 9. The band edges are predicted to be a mostly pure Fe-3*d* character, making the compound a Mott insulator. Further details and discussion on the effects of the *U*_eff_ parameter on the predicted properties of BaFeTeS are provided in the Supporting Information.

To further investigate whether the BaFeTeS phase assumes a Peierls distortion, or a charge-density-wave (CDW) arrangement, the investigation was expanded with noncollinear magnetic calculations, including spin–orbit coupling contributions. With respect to the structural considerations, the results showed no indication that the BaFeTeS analog exhibits any form of CDW or Peierls distortion. The most stable configuration converges toward a fully symmetrical arrangement of the Fe positions, regardless of spin orientation. It was, however, found that the magnetic interplane interaction energy is very small, with an energy contribution per formula unit of approximately 0.15 meV. Further details on the results of the noncollinear calculations are given in the Supporting Information.

## Discussion

Mössbauer spectra establish that BaFeTeS assumes no long-range magnetic order down to 6 K. Simultaneously, magnetic susceptibility measurements suggest that the compound is not in a Curie-paramagnetic state throughout the 2–300 K range either, despite the fact that an electronically insulating ground state is obvious by resistivity measurements. The mismatch between the DFT results predicting an ordered magnetic state and the experimental observations with respect to the magnetic behavior could originate from multiple sources: the DFT calculations could be overestimating the interplanar magnetic interactions, the origin of the experimental properties of BaFeTeS could be determined by a mechanism beyond the scope of DFT, or alternatively, it is also possible BaFeTeS orders at a temperature below 2 K. The latter is quite plausible, as the calculated interplane interaction energy is in fact comparable with the thermal energy of electrons around 2 K. The magnetocrystalline anisotropic Fe^2+^ ions would be expected to order due to diminishing geometric frustration, as compared to Co^2+^ ions; yet the opposite observation is made, which is counterintuitive.

The observed properties, along with the DFT calculations, imply that a plausible description for the magnetic properties of BaFeTeS includes a series of internally strongly coupled Fe-layers, extending parallel with the *ac*-plane. While internally ordered, spins in each of these individual planes may rotate independently of the spin alignment in the adjacent planes and thus assume no long-range spin ordering. This model explains the bulk of the magnetic behavior of BaFeTeS, and fully agrees with the high-temperature Mössbauer spectra; however, it fails to explain the broadening and asymmetry of the Mössbauer peaks at lower temperatures.

The electrical resistance measurements and correlation the results show with the Mössbauer spectra may provide some further insight into the nature of the effect, as the observations most likely originate from the same mechanism. As previously noted, the onset of the asymmetry and broadening in the Mössbauer spectra occurs together with a decrease in the effective band gap of the BaFeTeS phase. Further, the transition also involves the compound’s behavior changing from simple thermally activated charge carrier conductance to a different state, one where the electrical resistance increases at a slower rate with decreasing temperature than would be expected with simple thermal activation. Generally, this would indicate the presence of a second effect acting to decrease the resistance. In agreement with the indications from the Mössbauer data, the most likely cause would be some form of structural distortion, either static or dynamic.

While established models explaining Mössbauer asymmetry and broadening fail to convincingly explain the observed spectra, an alternative hypothesis could provide an explanation: a dynamic process based on a resonating valence bond (RVB) model.^44^ From the thermal displacement parameters of the SC-XRD refinement, it is concluded that Fe ions predominantly oscillate along the *a*-axis, parallel to the Fe–Te–Fe coupling. For simplicity, it is assumed the Fe–S–Fe coupling is rigid, and the full Fe–S chains oscillate synchronously. Furthermore, all atoms apart from Fe are considered static.

**Figure 10:**
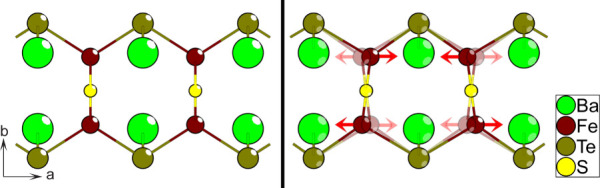
Primary mode of oscillation involved in the hypothetical dynamic process. The displacement is exaggerated for clarity, with the arrows showing the displacement axis.

If, from an initial state where all atoms are stationary, a single Fe–S chain is perturbed to start oscillating, the initially equal Fe(−Te)–Fe distances along the *a*-axis become unequal. This inequality subsequently affects the magnetic interaction energies. The Fe ions in the adjacent chains would then preferentially couple magnetically with the closer Fe, thereby attracting these Fe ions toward the initial chain, inducing a harmonic oscillation (Figure 10).

The adjacent chains would then oscillate at the same harmonic frequency as the initial chain, but with opposite phase, and this effect could then extend to the full 2D plane. However, the atomic oscillation cannot explain the Mössbauer spectra, because, in the Mössbauer time scale, the ions may be considered nearly stationary. Rather, at any given moment, the Fe ions throughout a polycrystalline powder sample (such as was utilized for the Mössbauer measurements) would be in a statistical distribution of local coupling environments, which the Mössbauer spectroscopy would randomly sample. In contrast to the slow atomic dynamics varying the local environment, the spin rotations are much faster and would prevent the Mössbauer data from exhibiting any clear hyperfine splitting.

The upper temperature limit of the effect would be determined by the thermal energy necessary for the movement of the ions to disrupt the coherent motion of adjacent chains. Above this temperature, the magnetic interaction between adjacent Fe–S chains is too weak to maintain coherent motion, thus resulting in the symmetric dipole observed in the high-temperature Mössbauer data between 220 and 293 K, characteristic of a state where electron spins rotate freely. At around 200 K, the strength of the magnetic coupling between the Fe ions along the *a*-axis becomes sufficient to induce coherent motion. At this temperature, the Fe ions experience the greatest ionic displacement, and by extension the greatest variance in the local environment, resulting in the broadest and most asymmetric Mössbauer peaks. As the temperature decreases below 200 K, the displacement of the Fe ions decreases. However, the strength of the magnetic coupling increases simultaneously, compensating for the decreased variance in spatial positions of the ions. As such, although the Fe ions exhibit less movement, the change in the local environment of the Fe ions for a given displacement becomes more significant. As a result, the Mössbauer dipole shifts toward a more symmetric arrangement as the temperature lowers, but the peaks remain broadened. This coupling model would also agree with the observation that the crystal structures with magnetic ions specifically exhibit particularly elongated thermal parameters along the *a*-axis.

There are previous reports in the literature of dynamics in low-dimensional systems with similar signatures in the temperature-dependent Mössbauer behavior. In the article referenced here by Hudson et al.,^38^ a Goldanskii-Karyagin-type asymmetry in the Mössbauer spectra is attributed to a dynamic effect where Fe^3+^ ions move between two adjacent sites. The dynamic effect is different from our case, and their observed Mössbauer spectra are not qualitatively identical, probably because of the differences in atomic lattices. Ultimately, this mechanism is a tentative explanation for what takes place in BaFeTeS. Settling this matter would necessitate further investigation, for instance by Raman spectroscopy on single crystals, which is a topic for future work.

## Conclusions

We synthesized single crystals of four structural analogs by solid-state synthesis from a nominal 1:1:1 ratio of BaS, Te, and the respective element Fe, Mn, Zn, or Ge. The crystal structures and nominal compositions were determined by SC-XRD and SEM/EDX analysis. All phases were additionally investigated by DFT analysis. The compounds assume a crystal structure described by the *Cmcm* space group. BaMnTeS was found to be telluride deficient, while the germanium analog exhibited a disordered half-occupancy BaGe_0.5_TeS. A relatively pure phase of BaFeTeS was obtained, which was analyzed by DC susceptibility, heat capacity, electrical resistance, and Mössbauer spectroscopy. The phase was found to undergo no first-order transitions and was found to remain magnetically disordered down to 6 K. Electrical resistance measurements of BaFeTeS exhibited two distinct phases, a broad intermediate state, and transitions at temperatures that correspond with features observed in temperature-dependent Mössbauer spectra. Mössbauer spectroscopy showed anomalous broadening and asymmetry that could not be readily explained by any well-known theory. A hypothesis, based on a dynamic resonating valence bond model, was proposed to explain the observations.

## Data Availability

Crystal information file (CIF) from the single crystal structure refinements are available free of charge (BaFeTeS_HT_Final.cif, BaFeTeS_LT_Final.cif, BaGeTeS_Final.cif, BaMnTeS_Final.cif, and BaZnTeS_Final.cif).

## Supporting Information Available

A file (Supporting Information.pdf) detailing extra information, which was omitted from the main manuscript, including: the refined atomic positions and thermal parameters of the four Ba*M*TeS phases; interatomic distances and angles of BaFeTeS; supplementary notes on the SC-XRD refinement and results Refinement details and parameters for Riedveld refinement of BaFeTeS PXRD; supplementary notes from the EDX analysis; *U*_eff_ dependence of the BaFeTeS band structure; details on the results with noncollinear calculations for BaFeTeS; and further details on the DFT results on the Mn, Zn, and Ge phases (PDF)
