# Supplementary material for: Synthesis and Properties of BaMTeS (M = Fe, Mn, Zn) and the Disordered Structural Analog BaGe0.5TeS
Source: Inorg Chem. 2024 Jun 6;63(24):10987–96. doi: 10.1021/acs.inorgchem.4c00146 (PMC11186005; doi:10.1021/acs.inorgchem.4c00146)
Supplement: Supplementary file 1 — ic4c00146_si_001.pdf [file ic4c00146_si_001.pdf]

Supporting Information to:

Synthesis and Properties of Ba*M*TeS (*M* = Fe, Mn, Zn) and the Disordered Structural Analog $BaGe_{0.5}TeS$

Authors: Emil H. Frøen[1], Domenic Nowak[2], Peter Adler[3], Martin Valldor[1]*

[1]Centre for Materials Science and Nanotechnology (SMN), Department of Chemistry, University of Oslo, Sem Sælands vei 26, N-0371 Oslo, Norway

[2]Leibniz Institute for Solid State and Materials Research (IFW), Helmholtzstraße 20, 01069 Dresden, Germany

[3]Max Planck Institute for Chemical Physics of Solids, Nöthnitzer Straße 40, 01187 Dresden, Germany

*The corresponding author e-mail: e.h.froen@smn.uio.no

The atomic positions given here are not the same as those given in the respective CIF files; the given position of each atom is chosen to be equivalent across the phases for ease of comparison.

Table S1: Atomic positions and isotropic thermal parameters of BaFeTeS, measured at room temperature.

Atom	Site	*x*	*y*	*z*	U_{iso} (Å^2)
Ba	4*c*	0	0.11236(9)	¼	0.0123(5)
Fe	4*c*	0	0.3964(2)	¼	0.017(2)
Te	4*c*	½	0.2994(2)	¼	0.0192(6)
S	4*b*	0	½	0	0.013(2)

Table S2: Atomic positions and isotropic thermal parameters of BaFeTeS, measured at 100 K.

Atom	Site	*x*	*y*	*z*	U_{iso} (Å^2)
Ba	4*c*	0	0.11252(2)	¼	0.00473(8)
Fe	4*c*	0	0.39669(6)	¼	0.0070(2)
Te	4*c*	½	0.29898(3)	¼	0.00646(9)
S	4b	0	½	0	0.0051(3)

Table S3: Atomic positions and isotropic thermal parameters of BaMnTeS, measured at room temperature.

Atom	Site	*x*	*y*	*z*	Occupancy	U_{iso} (Å^2)
Ba	4*c*	0	0.10894(2)	¼	1	0.0178(2)
Mn	4*c*	0	0.39073(6)	¼	1	0.0199(3)
Te	4*c*	½	0.29216(3)	¼	0.86	0.0200(2)
S1	4*b*	0	½	0	1	0.0169(3)
S2	4*c*	½	0.29216(3)	¼	0.14	0.0200(2)

Table S4: Atomic positions and isotropic thermal parameters of BaZnTeS, measured at room temperature.

Atom	Site	*x*	*y*	*z*	U_{iso} (Å^2)
Ba	4*c*	0	0.10766(6)	¼	0.0121(3)
Zn	4*c*	0	0.3926(2)	¼	0.0145(6)
Te	4*c*	½	0.29667(8)	¼	0.0173(4)
S	4b	0	½	0	0.0134(2)

Table S5: Atomic positions and isotropic thermal parameters of $BaGe_{0.5}TeS$, measured at room temperature.

Atom	Site	*x*	*y*	*z*	Occupancy	U_{iso} (Å^2)
Ba	4*c*	0	0.10596(3)	¼	1	0.0137(2)
Ge	4*c*	0	0.3916(2)	¼	½	0.0111(4)
Te	4*c*	½	0.30632(4)	¼	1	0.0172(2)
S	4*b*	0	½	0	1	0.0162(5)

Table S6: Interatomic distances within the BaFeTeS crystal structure at room temperature, as determined by SC-XRD.

Atomic positions	Distance (Å)
Ba – Te	3.4849(5)
Ba – S	3.2781(3)
Mn – Te	2.6653(6)
Mn – S	2.3897(6)

Table S7: Interatomic angles within the BaFeTeS crystal structure at room temperature, as determined by SC-XRD.

Atomic Angle	Angle (°)
Te1(i)-Ba1-Te1	80.31(1)
Te1(i)-Ba1-S1(ii)	85.819(6)
Te1(i)-Ba1-S1(iii)	144.226(5)
Te1(i)-Ba1-S1(iv)	85.819(6)
Te1(i)-Ba1-S1(v)	144.226(5)
Te1-Ba1-S1(ii)	144.226(5)
Te1-Ba1-S1(iii)	85.819(6)
Te1-Ba1-S1(iv)	144.226(5)
Te1-Ba1-S1(v)	85.819(6)
S1(ii)-Ba1-S1(iii)	86.561(7)
S1(ii)-Ba1-S1(iv)	65.998(6)
S1(ii)-Ba1-S1(v)	122.22(1)

S1(iii)-Ba1-S1(iv)	122.22(1)
S1(iii)-Ba1-S1(v)	65.998(6)
S1(iv)-Ba1-S1(v)	86.561(7)
Ba1-Te1-Ba1(vi)	80.31(2)
Ba1-Te1-Mn1	82.36(2)
Ba1-Te1-Mn1(vi)	162.68(2)
Ba1(vi)-Te1-Mn1	162.68(2)
Ba1(vi)-Te1-Mn1(vi)	82.36(2)
Mn1-Te1-Mn1(vi)	114.96(3)
Te1(i)-Mn1-Te1	114.96(3)
Te1(i)-Mn1-S1	110.938(6)
Te1(i)-Mn1-S1(vii)	110.938(6)
Te1-Mn1-S1	110.938(6)
Te1-Mn1-S1(vii)	110.938(6)
S1-Mn1-S1(vii)	96.68(3)
Ba1(viii)-S1-Ba1(ix)	86.561(6)
Ba1(viii)-S1-Ba1(x)	93.439(6)
Ba1(viii)-S1-Ba1(xi)	180
Ba1(viii)-S1-Mn1	85.08(2)
Ba1(viii)-S1-Mn1(xii)	94.92(2)
Ba1(ix)-S1-Ba1(x)	180
Ba1(ix)-S1-Ba1(xi)	93.439(6)
Ba1(ix)-S1-Mn1	85.08(2)
Ba1(ix)-S1-Mn1(xii)	94.92(2)
Ba1(x)-S1-Ba1(xi)	86.561(6)
Ba1(x)-S1-Mn1	94.92(2)
Ba1(x)-S1-Mn1(xii)	85.08(2)
Ba1(xi)-S1-Mn1	94.92(2)
Ba1(xi)-S1-Mn1(xii)	85.08(2)
Mn1-S1-Mn1(xii)	180

(i)	x-1,y,z
(ii)	x-1/2,y-1/2,z
(iii)	x+1/2,y-1/2,z

(iv)	-x-1/2,-y+1/2,z+1/2
(v)	-x+1/2,-y+1/2,z+1/2
(vi)	x+1,y,z
(vii)	-x,-y+1,z+1/2
(viii)	x-1/2,y+1/2,z
(ix)	x+1/2,y+1/2,z
(x)	-x-1/2,-y+1/2,z-1/2
(xi)	-x+1/2,-y+1/2,z-1/2
(xii)	-x,-y+1,z-1/2

SUPPLEMENTARY NOTES – SC-XRD REFINEMENT

Refining the occupancies of Te for the Fe, Zn and Ge analogs all result in some reduced occupancy, ranging from 4-9%, but unlike the Mn analog, refining the Te occupancy does not result in a major improvement of the refinement statistics.

The half-occupied Ge-analog has a significantly divergent *b*-axis lattice parameter, compared with the other, here presented, structural homologs. The possibility of the compound exhibiting an ordered arrangement of Ge occupancies with a space group such as $P2_1/m$ was considered, but no satisfactory refinement of the data corresponding to such a structure was obtained.

RIETVELD REFINEMENT – REFINEMENT DETAILS

The background correction used Legendre polynomials with 15 terms, while the peak profile fitting used the Pseudo-Voigt function employing the GU, GW, LX and LY parameters. A Berar-Baldinozzi asymmetry correction with four terms was applied.

Table S8: Refinement parameters for Rietveld refinement of BaFeTeS

Formula	BaFeTeS
Radiation	Cu *K*α (λ = 1.540593 Å)
Instrument	Bruker D8 Discover
Crystal System	Orthorhombic
Space Group	Cmcm (No. 63)

Physical Appearance	Black
Temperature/K	293
Formula Weight/g mol^{-1}	352.85
a/Å	4.45517(3)
b/Å	14.26727(9)
c/Å	7.09845(4)
V/Å^3	451.1996
Z	4
ρ_{calc}/g cm^{-3}	5.1943
R1 (obs)/%	6.44
R1 (all)/%	7.51
wR2 (obs)/%	7.52
wR2 (all)/%	7.71

Table S9: Atomic parameters for Rietveld refinement of BaFeTeS

	x	y	z	Uiso
Ba	0	0.3877(2)	0.25	0.0081(8)
Fe	0	0.1053(3)	0.25	0.019(2)
Te	0.5	0.1992(2)	0.25	0.021(1)
S	0	0	0	0.002(1)

MÖSSBAUER SPECTROSCOPY

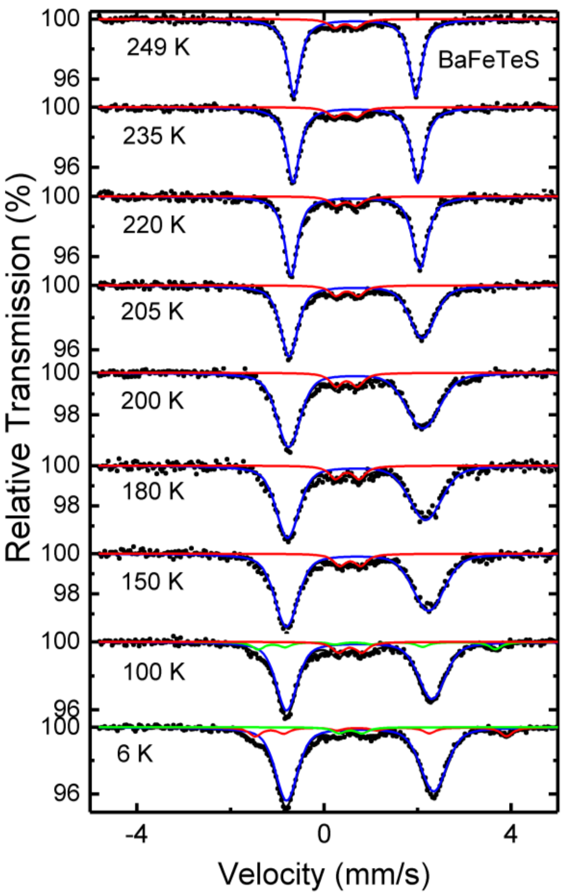

Figure S1. Alternate fitting of the temperature dependent Mössbauer spectra, assuming a correlated distribution in IS and QS. Dots, black lines and colored lines correspond to the experimental data, the calculated spectra, and the component spectra, respectively. The small signals are attributed to impurities.

SUPPLEMENTARY OBSERVATIONS – EDX

A systematic oddity was noticed during the EDX analysis of $BaGe_{0.5}TeS$, where the X-ray signal originating from germanium would, seemingly arbitrarily, be significantly reduced compared with the expected 0.5 stoichiometry when measuring at certain spots of a given crystal. The signal would suggest roughly a 0.25 stoichiometry of Ge instead, and consistently the same halved value. Measuring elsewhere on the same crystal there would always be a spot which would yield the "0.5" composition. The precise conditions for obtaining this halved signal are unknown, but it was observed for several different crystals.

DFT ANALYSIS – Mn, Zn and Ge

For the Fe and Mn analogs, the $U_{\text{eff}} = 0$ eV provide the best structural match with respect to lattice parameters, the unit cell becoming larger with increased U_{eff}. Conversely, the lattice parameters of the Zn analog match the best at $U_{\text{eff}} = 6$ eV, with the unit cell shrinking with lower U_{eff}.

The Mn analog is consistently predicted to be a direct semiconductor, with a gamma point transition for all values of U_{eff}. The nature of the transition varies significantly, however. At low U_{eff}, the VBM is dominated by Mn-3*d* states, but highly hybridized with Te and S states. The CBM is dominated by Mn-3*d* states. At $U_{\text{eff}} = 2$ eV, the nature of the VBM transitions to being dominated by Te-5*p* states, shifting further towards a pure Te-5*p* state with higher values of U_{eff}. With increasing U_{eff}, the CBM shifts towards a a hybrid of Ba-6*s* and Mn-3*d* states, making the compound shift from a Mott- to a charge-transfer insulator.

The width of the band gap of the Zn analog varies relatively little with a U_{eff} range of 0-6 eV, increasing from 1.20 to 1.33 eV. At lower values of U_{eff}, the band gap is predicted to have a direct gamma-point transition. U_{eff} values of 4 eV or higher, the character changes to an indirect Γ-Y transition. The character of the band edges remain qualitatively unchanged with variation in U_{eff}. The VBM consists primarily of Te-5*p* states, while the CBM is a hybrid of states from all elements in the structure.

The Ge analog exhibits an indirect Γ-A transition of 0.93 eV. The VBM consists primarily of Te-5*p* states, while the CBM is a hybrid of Te-5*p* and Ge-4*s* states.

BAND STRUCTURE OF BaFeTeS – DETAILS OF U_{eff} DEPENDENCE

For BaFeTeS, a considerable band gap only appears with U_{eff} of 2 eV or greater. Considering the experimentally determined band gap from the electric resistance, U_{eff} values of 0-1 eV thus provide the closest match with the experimental expectation. In addition to the width, the nature of the band gap varies as well; $U_{\text{eff}} = 0$-3 eV results in an indirect transition, while larger values 5-6 eV result in a direct Gamma-point transition. $U_{\text{eff}} = 4$ eV results in an intermediate state. At $U_{\text{eff}} = 0$, the band structure exhibits a protrusion in both the conduction band minimum (CBM) and valence band maximum (VBM) bands, but it appears continuous at all points and is not a van Hove singularity. With increasing values of U_{eff}, this feature broadens out to assume a relatively broad band. At lower values of U_{eff}, the band edges are predicted to be a mostly

pure Fe-3*d* character, making the compound a Mott-insulator up to U_{eff} = 2 eV. Increasing U_{eff} beyond this point, the VBM character transitions to a pure Te-5*p* character, making the compound a charge-transfer compound.

NONCOLLINEAR MAGNETIC STRUCTURE CALCULATIONS FOR BaFeTeS

A range of noncollinear magnetic configurations were attempted in order to replicate a state corresponding with the experimentally observed lack of ordering at low temperatures. These were all found to be higher-energy states than the simple collinear antiferromagnetic arrangement, but the magnetic coupling energy between adjacent Fe-planes was found to be marginal: Aligning adjacent planes to have orthogonal AFM spin configurations, at U_{eff} = 0 eV, the energy is about ~0.15 meV per formula unit higher than the most favorable fully collinear arrangement; this is with the common tendency of DFT to greatly overestimate magnetic interaction energies. Applying U_{eff} = 3 eV, the interaction energy falls to ~0.05 meV per formula unit. For comparison, the energy increase involved in breaking the planar AFM arrangement ranges from ~16 to over 100 meV per formula unit, depending on what anti-parallel arrangements are broken, with the Fe – S – Fe couplings being the stronger of the two intra-planar couplings. The magnitude of the band gaps of these configurations were found to be similar to the nominally most favorable antiferromagnetic arrangement, and the qualitative character of the band edges remained unchanged, although the band structures of the non-collinear structures were not extensively investigated.

While the most favorable magnetic arrangement was collinear, equivalent with what was found for the collinear calculations, the results indicate an anisotropy in the orientation of the spin, although the precise details are dependent on the U_{eff} value. At U_{eff} = 3 eV, the lowest energy state arranges the spin parallel with the *a*-axis, that is, parallel with the Fe – Te – Fe coupling axis. At U_{eff} = 0 eV, the lowest energy state places the spin parallel with the *b*-axis instead. In both of these cases, the remaining two spin orientations are nearly isotropic.
